# Seasonality and other risk factors for fleas infestations in domestic dogs and cats

**DOI:** 10.1111/mve.12636

**Published:** 2023-01-09

**Authors:** Sean Farrell, John McGarry, Peter‐John Mäntylä Noble, Gina J. Pinchbeck, Sophie Cantwell, Alan D. Radford, David A. Singleton

**Affiliations:** ^1^ School of Biosciences University of Kent Canterbury Kent UK; ^2^ Institute of Infection, Veterinary and Ecological Sciences University of Liverpool Neston UK

**Keywords:** companion animals, electronic health records, epidemiology, fleas, Siphonaptera

## Abstract

Fleas in the genus *Ctenocephalides* are the most clinically important parasitic arthropods of dogs and cats worldwide yet risk factors that might increase the risk of infestation in small animals remains unclear. Here we developed a supervised text mining approach analysing key aspects of flea epidemiology using electronic health records from domestic cats and dogs seen at a sentinel network of 191 voluntary veterinary practices across Great Britain between March 2014 and July 2020. Our methods identified fleas as likely to have been present during 22,276 of 1,902,016 cat consultations (1.17%) and 12,168 of 4,844,850 dog consultations (0.25%). Multivariable logistic regression modelling found that animals originating from areas of least deprivation were associated with 50% reductions in odds of veterinary‐recorded flea infestation compared to the most deprived regions in England. Age of the animal was significantly associated with flea presentation in both cats and dogs, with cases peaking before animals reached 12 months. Cases were recorded through each study years, peaking between July and October, with fluctuations between each year. Our findings can be used towards healthcare messaging for veterinary practitioners and owners.

## Graphical abstract

The greatest risk for flea infestation occurs for both dogs and cats below the age of 1 year. Small terriers and toy dog breeds have double the odds for flea infestation compared to Retrievers; in cats Asian breeds had half the odds compared to Western European breeds. Areas of increased deprivation saw increased odds for flea infestation, suggesting strong socioeconomic challenges to flea treatment routines

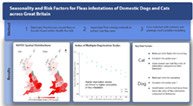

## INTRODUCTION

There are 2500 species and sub‐species of *Siphonaptera* flea across 18 families and 220 genera worldwide (Lewis, 1998), but within the sphere of companion animal veterinary medicine, just two of the cat flea *Ctenocephalides felis* (Bouche, 1835) (Siphonaptera: Pulicidae), and to a lesser extent the dog flea *Ctenocephalides canis* (Curtis, 1826) (Siphonaptera: Pulicidae), are of major health significance. Fleas are the most commonly seen ectoparasite in companion animal practice (Farkas et al., 2009), infesting homes and posing a considerable nuisance factor to owners. They are often associated with intense animal discomfort and flea allergy dermatitis and can act as vectors for several important zoonotic diseases (Florin et al., 2008; Lam & Yu, 2009; Pérez‐Osorio et al., 2008).

Cat fleas are a cause of severe irritation and allergic dermatitis in susceptible, sensitized hosts causing allergic dermatitis, one of the most important dermatological conditions seen in small animal veterinary practices (Lam & Yu, 2009). Besides this, fleas bite people and can be vectors of zoonotic pathogens, such as Bartonella *spp*. and Rickettsia *felis* (Bai et al., 2017; Leulmi et al., 2014). *Ctenocephalides felis* readily completes its life cycle feeding on pets within homes or in peri‐domestic settings where temperature and humidity conditions are suitable to support the immature flea stages in the environment (Cooper et al., 2020; Halos et al., 2014; Rust, 2017). This species does not appear to be particularly host‐specific; it is suggested that populations may be maintained in wildlife (Clark et al., 2018) which, in part, may explain its persistence in domestic infestations. There is a growing discussion about the roles of flea treatment with regards to the environmental impact they may have, following the recent demonstration of environmentally damaging levels of imidacloprid and fipronil residues in British rivers (Perkins et al., 2021).

There has been a large amount of previous research on domestic animal flea biology and control over the past 20 years such as a 2005 study across 31 UK veterinary practices that found flea infestations of 21.09% in cats and 6.82% in dogs finding increased infestations where a cat was present within a household with other pets (Bond et al., 2007). The overwhelming flea species across both cats and dogs was determined to be *C. felis* accounting for 98.83% in cats and 93.15% in dogs. A similar study in Hungary examining 13 veterinary practices found that 22.9% of cats and 14.1% in dogs were flea infested, and another study conducted in Italy across four veterinary practices reported 17.9% of dogs to be flea infested, also finding correlations between infestation and living with other dogs and cats (Farkas et al., 2009; Rinaldi et al., 2007). Recently, a national practice‐level survey completed in the UK involving 326 premises conducted examinations of 812 cats and 662 dogs between April and June 2018, of which they found 28.1% and 14.4% were infested with fleas respectively, and that 90% of recovered fleas was *C. felis* (Abdullah et al., 2019). A survey‐based study showed statistically significant geographical variation, including a significant decline in prevalence from south to north, but none of the animal factors investigated (breed, sex, neutered status, or whether the pet had been abroad) showed any relationship with the underlying geographical distribution (Cooper et al., 2020). Veterinary practice‐level surveys generate important baseline information towards our understanding of the epidemiology of flea‐associated disease, but interpretation of data is limited by the relatively small samples taken within a short period of time.

Many variables that may potentially lead to infestation and reinfestation of domestic pets remain undefined, in part because research aimed at exploring epidemiological risk factors often necessitates large‐scale sampling and extensive work recruiting veterinarians, pets and owners. Veterinary electronic health records (EHRs) provide a near real‐time capture of recorded events providing an opportunity to explore conditions whose epidemiology is not entirely understood. The aim of this study is to use EHR data from a sentinel network of veterinary practices from across Great Britain (GB) over a six‐year period to develop a text mining technique to identify at scale flea infestations recorded by veterinary professionals to assess the impact of season; geographical spatial distribution; pet breed; age; sex; and neutered status associated with flea cases diagnosis.

## MATERIAL AND METHODS

### Data extraction and inclusion criteria

Veterinary EHRs were collected between March 2014 and July 2020 from a sentinel network of 191 volunteer veterinary practices across GB; a full description of the Small Animal Veterinary Surveillance Network (SAVSNET), has been presented elsewhere (Sánchez‐Vizcaíno et al., 2017). Briefly, veterinary practices using practice management software previously made compatible with SAVSNET data exchange were recruited based on convenience. In participating practices, data is collected from each booked consultation (where an owner has made an appointment to see a veterinary surgeon or nurse). Owners attending participating practices are given the option to opt out at the time of their consultation, thereby excluding their data. For those that participate, data is collected on a consultation‐by‐consultation basis and can include information about the animal (e.g., species, breed, sex, neuter status, age, owner's postcode, insurance, and microchipping status), as well as a free‐text clinical narrative, treatments dispensed, and the vaccination history. SAVSNET has ethical approval from the University of Liverpool Research Ethics Committee (RETH000964).

For the exploration of data used in this study, a case was first defined as an animal that presented with fleas or flea dirt at the time of consultation, as observed and record by the attending practitioner within the free‐text clinical narrative where no other conflicting diagnosis was made. Putative cases were provisionally identified by screening all clinical narratives using a Python regular expression (Regex) for the identification of consultations where fleas or flea dirt were recorded matching our case definition.

**FIGURE 1:**
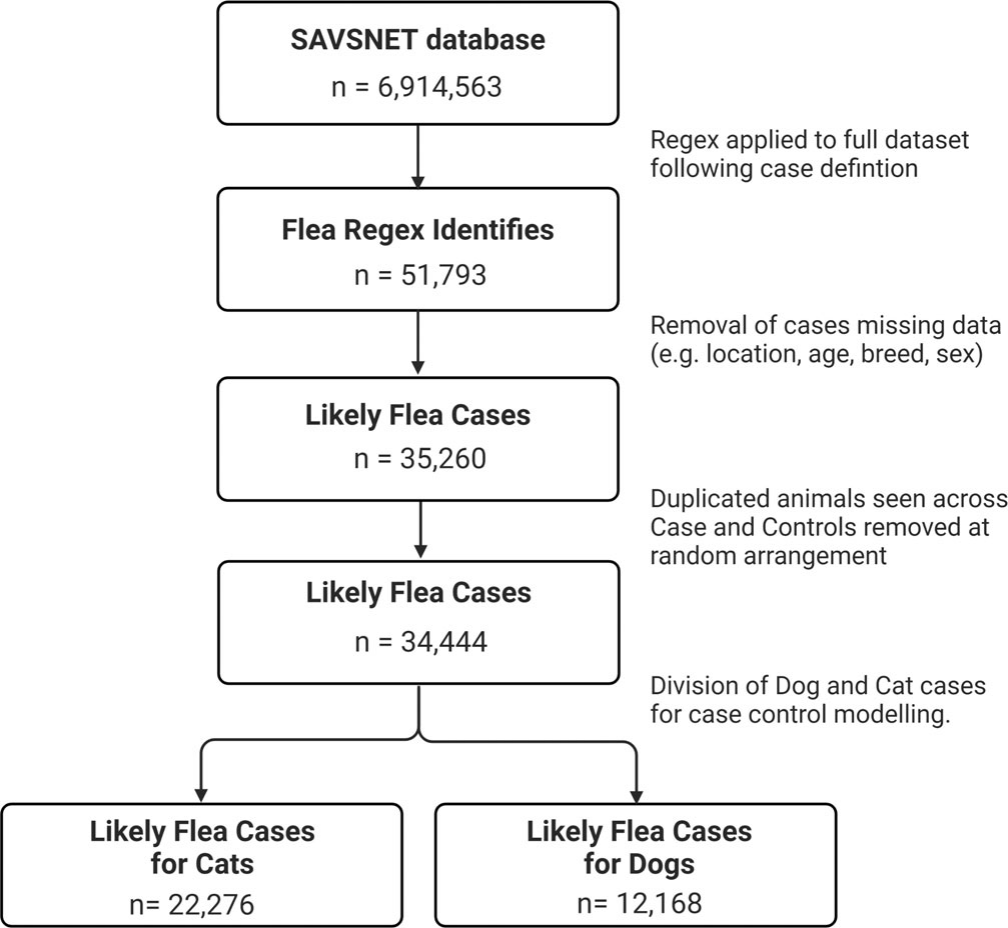
Data mining pathway used to extract flea consultations from the SAVSNET electronic health record dataset, with inclusion of data cleaning stages before splitting on species.

The regex was developed iteratively, each time using a new random sample of 10,000 consultations selected from the entire SAVSNET database to provide phrases and spelling variations to better improve the accuracy of the regex in finding cases that match our case definition. For each iteration, up to 100 random identified records were manually read by one of the authors (SF) to identify new terms requiring inclusion of exclusion. Examples of common negations are with reference only to flea treatments; general flea‐related advice; owner diagnosis of fleas; or previous infestations all of which was explicitly independent of fleas or flea dirt presence at the time of the consultation. This process was repeated until consistent true positive cases dominated these 100 random records and new negations were rare, before being applied to the entire SAVSNET dataset; any identified EHRs were at this point regarded as true cases. This process is outlined in Figure 1 with the final regex accessible in the supplementary information.

### Animal data

In order to determine risk factors associated with recorded flea infestation, we conducted a retrospective case–control study. Control consultations (set at a ratio of one case to every six controls) were randomly chosen from the database where it did not match our regex produced to capture flea cases. For each case and control, date of birth, date of consultation, owner's postcode, sex, neuter status, species, breed, and the clinical narrative were collected. Where any case or control was missing any data (most frequently this was geographical or age data), they were removed from the study. When an animal was seen multiple times, either within the case or control population, or between them, duplicate animal consultation records were removed at random leaving one consultation record per animal. Once the final dataset had been attained, 100 consultations were randomly extracted and given to a separate author (DS) to manually verify the success of the extraction process. Whether a consultation was a case or control was hidden from the reviewer and only revealed once the manual reading was complete and for statistical metrics to be determined.

To allow for investigation of breed as a risk factor, breeds were categorized according to genetic markers, as identified by the VonHoldt and Lipinski in separate breed‐based genetic‐associated papers (Lipinski et al., 2008; Vonholdt et al., 2010). This classification system organizes canine breeds into one of 11 genetic types (with an additional unclassified category, such as retriever, spaniel, and small terriers). We also include a ‘crossbreed’ breed type. For cats, the breeds were associated into four types based on regions of origin, Asian, Mediterranean, West European and crossbreed, with an additional unclassified group.

Owner postcodes were used to calculate Nomenclature of Territorial Units for Statistics (NUTS) 1 codes. Data on the degree of urbanization for each postcode also were obtained using a 10‐fold score for England and Wales and 7‐fold score for Scotland (Bibby, 2013; Scottish Government, 2016). Regions 1 and 2 in Scotland, and A, B and C in England and Wales were classified as urban locations, with the remaining classified as rural. Records from postcodes were converted to lower super output areas (LSO) if located with England and were paired with multiple deprivation values using the Index of Multiple Deprivation (IMD) Decile 2019, where decile 1 represents the most deprived 10% of LSOAs, decreasing to the least deprived 10% of LSOAs in decile 10 (UK Government, 2019).

### Statistical analysis

Initial quantitative analysis was used to identify broader features of the dataset such as Kendall's т coefficient test to verify presence of correlation between number of cats against the number of dog cases per 100 consultations per surveyed practice. Univariate mixed effect logistic regression was conducted utilizing case–control status as a binary dependent variable. Every explanatory variable (Tables 1 and 2) was explored, with likelihood ratio test (LRT chi‐squared test) used to assess fit compared to a null model, using practice as a random effect.

**TABLE 1:** Risk of veterinary‐recorded flea infestation in dogs (*N* = 12,168) from multivariable logistic regression model throughout Great Britain.

Variable	Level	Case (%)	Controls (%)	Beta	SE^a^	OR^b^ (95% CI^c^)	*p*
	Intercept			−3.843	0.113	–	–
Categorical variables							
Sex + Neuter	Male entire	3264 (26.8)	31,029 (19.7)	–	–	1.00	–
	Male neutered	3122 (25.7)	49,660 (31.4)	0.603	0.027	0.55 (0.52–0.58)	<0.001
	Female entire	2875 (23.6)	25,965 (16.4)	0.049	0.028	1.05 (0.99–1.11)	0.078
	Female neutered	2906 (23.9)	51,249 (32.5)	−0.680	0.028	0.51 (0.48–0.54)	<0.001
Months	January	726 (6)	13,927 (8.8)	–	–	1.00	–
	February	650 (5.3)	13,353 (8.5)	0.061	0.056	0.94 (0.84–1.05)	0.272
	March	453 (3.7)	13,125 (8.3)	0.415	0.061	0.66 (0.59–0.75)	<0.001
	April	418 (3.4)	11,122 (7)	0.325	0.063	0.72 (0.64–0.82)	<0.001
	May	439 (3.6)	11,631 (7.4)	0.331	0.062	0.72 (0.64–0.81)	<0.001
	June	656 (5.4)	12,764 (8.1)	0.021	0.056	0.98 (0.88–1.09)	0.703
	July	1406 (11.6)	14,963 (9.5)	0.581	0.048	1.79 (1.63–1.96)	<0.001
	August	1698 (14)	13,970 (8.8)	0.838	0.046	2.31 (2.11–2.53)	<0.001
	September	1846 (15.2)	13,638 (8.6)	0.940	0.046	2.56 (2.34–2.80)	<0.001
	October	1708 (14)	14,145 (9)	0.832	0.046	2.30 (2.10–2.52)	<0.001
	November	1288 (10.6)	13,061 (8.3)	0.640	0.048	1.90 (1.72–2.09)	<0.001
	December	879 (7.2)	12,204 (7.7)	0.327	0.052	1.39 (1.25–1.54)	<0.001
Rural/Urban	Rural	3515 (28.9)	51,184 (32.4)	–	–	1.00	–
	Urban	8653 (71.1)	106,719 (67.6)	0.155	0.026	1.17 (1.11–1.23)	<0.001
NUTS1	UKC (North East)	704 (5.8)	14,337 (9.1)	–	–	1.00	–
	UKD (North West)	1328 (10.9)	16,167 (10.2)	0.528	0.120	1.70 (1.34–2.15)	<0.001
	UKE (Yorks & Humber)	1157 (9.5)	17,843 (11.3)	0.422	0.123	1.53 (1.20–1.94)	0.001
	UKF (East Midlands)	637 (5.2)	9797 (6.2)	0.185	0.129	1.20 (0.93–1.55)	0.153
	UKG (West Midlands)	699 (5.7)	11,937 (7.6)	0.142	0.133	1.15 (0.89–1.50)	0.287
	UKH (East of England)	1691 (13.9)	19,295 (12.2)	0.571	0.124	1.77 (1.39–2.26)	<0.001
	UKI (Greater London)	110 (0.9)	1708 (1.1)	0.336	0.171	1.40 (1.00–1.96)	0.049
	UKJ (South East)	2763 (22.7)	32,446 (20.5)	0.592	0.112	1.81 (1.45–2.25)	<0.001
	UKK (South West)	1891 (15.5)	18,307 (11.6)	0.813	0.121	2.26 (1.78–2.86)	<0.001
	UKL (Wales)	885 (7.3)	7913 (5)	0.748	0.141	2.11 (1.60–2.79)	<0.001
	UKM (Scotland)	302 (2.5)	8153 (5.2)	0.272	0.121	0.76 (0.60–0.97)	0.025
Breed	Retriever	122 (1)	2041 (1.3)	–	–	1.00	–
	Ancient spitz	3322 (27.3)	33,811 (21.4)	0.318	0.101	1.37 (1.13–1.68)	0.002
	Crossbreed	619 (5.1)	6823 (4.3)	0.881	0.041	2.41 (2.23–2.62)	<0.001
	Herding	642 (5.3)	14,699 (9.3)	0.817	0.056	2.26 (2.03–2.53)	<0.001
	Mastiff like	789 (6.5)	19,339 (12.2)	0.016	0.055	0.98 (0.88–1.10)	0.766
	Scent hound	178 (1.5)	3889 (2.5)	0.072	0.086	1.07 (0.91–1.27)	0.403
	Sight hound	112 (0.9)	2456 (1.6)	0.143	0.104	1.15 (0.94–1.42)	0.170
	Small terriers	1967 (16.2)	17,092 (10.8)	1.039	0.044	2.83 (2.59–3.08)	<0.001
	Spaniel	1464 (12)	18,705 (11.8)	0.632	0.046	1.88 (1.70–2.06)	<0.001
	Toy	1132 (9.3)	11,150 (7.1)	0.851	0.049	2.34 (2.13–2.58)	<0.001
	Unclassified	1462 (12)	21,774 (13.8)	0.527	0.046	1.69 (1.55–1.85)	<0.001
	Working dog	358 (2.9)	6124 (3.9)	0.337	0.066	1.40 (1.23–1.60)	<0.001
Continuous variables							
Age	Linear fit	–	–	0.102	0.020	1.11 (1.06–1.15)	<0.001
	Quadratic fit	–	–	0.052	0.014	1.05 (1.03–1.08)	<0.001
	Cubic fit	–	–	0.068	0.010	0.93 (0.92–0.95)	<0.001

^a^ Standard error.

^b^ odds ratio.

^c^ 95% confidence interval.

**TABLE 2:** Risk of veterinary‐recorded flea infestation in cats (*N* = 22,276) from multivariable logistic regression model throughout Great Britain.

Variable	Level	Case (%)	Control (%)	Beta	se^a^	OR^b^ (95% CI^c^)	*p*
	Intercept			−2.321	0.104	0.098	<0.001
Categorical variables							
Sex + Neuter	Male entire	2993 (13.4)	2993 (7.6)	–	–	1.00	–
	Male neutered	7890 (35.4)	7890 (41.1)	−0.575	0.027	0.56 (0.53–0.59)	<0.001
	Female entire	3369 (15.1)	3369 (9.1)	−0.044	0.031	0.96 (0.90–1.02)	0.155
	Female neutered	8015 (35.9)	8015 (42.2)	−0.576	0.027	0.56 (0.53–0.59)	<0.001
Months	January	1862 (8.4)	8806 (9.0)	–	–	1.00	–
	February	1513 (6.8)	8161 (8.3)	−0.119	0.039	0.89 (0.82–0.96)	0.002
	March	1239 (5.6)	7779 (7.9)	−0.207	0.041	0.81 (0.75–0.88)	<0.001
	April	980 (4.4)	6565 (6.7)	−0.258	0.044	0.77 (0.71–0.84)	<0.001
	May	989 (4.4)	6872 (7.0)	−0.333	0.044	0.72 (0.66–0.78)	<0.001
	June	1324 (5.9)	7637 (7.8)	−0.219	0.040	0.80 (0.74–0.87)	<0.001
	July	2190 (9.8)	8843 (9.0)	0.135	0.036	1.14 (1.07–1.23)	<0.001
	August	2442 (11.0)	8646 (8.8)	0.265	0.035	1.30 (1.22–1.40)	<0.001
	September	2614 (11.7)	8609 (8.8)	0.339	0.035	1.40 (1.31–1.50)	<0.001
	October	2825 (12.6)	9386 (9.6)	0.317	0.034	1.37 (1.28–1.47)	<0.001
	November	2480 (11.1)	8796 (9.0)	0.277	0.035	1.32 (1.23–1.41)	<0.001
	December	1809 (8.1)	7874 (8.0)	0.096	0.038	1.10 (1.02–1.19)	0.011
Rural/Urban	Rural	5030 (22.5)	26,824 (27.3)	–	–	1.00	–
	Urban	17,237(77.4)	71,150 (72.6)	0.078	0.021	1.08 (1.04–1.13)	<0.001
NUTS1	UKC (North East)	821 (3.7)	4799 (4.9)	–	–	1.00	–
	UKD (North West)	2420 (10.8)	8277 (8.4)	0.395	0.073	1.48 (1.29–1.71)	<0.001
	UKE (Yorks and Humber)	2076 (9.3)	8848 (9.0)	0.212	0.072	1.24 (1.07–1.42)	0.003
	UKF (East Midlands)	1194 (5.4)	5236 (5.3)	0.339	0.081	1.40 (1.20–1.65)	<0.001
	UKG (West Midlands)	1530 (6.9)	5962 (6.1)	0.066	0.085	1.07 (0.90–1.26)	0.437
	UKH (East of England)	3053 (13.7)	11,887 (12.1)	0.425	0.071	1.53 (1.33–1.76)	<0.001
	UKI (Greater London)	402 (1.8)	1229 (1.3)	0.296	0.115	1.34 (1.07–1.68)	0.010
	UKJ (South East)	5924 (26.6)	26,348 (26.8)	0.234	0.066	1.26 (1.11–1.44)	<0.001
	UKK (South West)	3233 (14.5)	12,114 (12.3)	0.316	0.072	1.37 (1.19–1.58)	<0.001
	UKL (Wales)	1079 (4.8)	6795 (6.9)	−0.581	0.090	0.56 (0.47–0.67)	<0.001
	UKM (Scotland)	535 (2.4)	6479 (6.6)	−1.088	0.124	0.34 (0.26–0.43)	<0.001
Breed	West Europe	1075 (4.8)	5398 (5.5)	–	–	1.00	–
	Asian	254 (1.1)	3000 (3.1)	−0.610	0.077	0.54 (0.47–0.63)	<0.001
	Crossbreed	20,469 (91.9)	86,941 (88.7)	0.187	0.037	1.12 (1.12–1.30)	<0.001
	Mediterranean	6 (0.02)	49 (0.05)	−0.207	0.466	0.83 (0.33–2.03)	0.657
	Unclassified	463 (2.0)	2586 (2.6)	−0.263	0.064	1.77 (0.68–0.87)	<0.001
Continuous variables							
Age	Linear fit	–	–	−0.042	0.018	0.96 (0.93–0.99)	0.016
	Quadratic fit	–	–	0.099	0.011	1.10 (1.08–1.13)	<0.001
	Cubic fit	–	–	0.101	0.009	0.90 (0.89–0.92)	<0.001

^a^ Standard error.

^b^ odds ratio.

^c^ 95% confidence interval.

Continuous explanatory variables were assessed to ascertain presence (or absence) of a clear linear relationship with the dependent variable and those displaying non‐linear relationships were fitted with polynomials, up to cubic fits, depending on which provided best fit via an LRT and visualized with sjPlot (Daniel Lüdecke, 2019). Explanatory variables with an LRT of *p* ≤ 0.2 compared to the null model were included in an initial multivariable logistic regression model. A backwards selection process was utilized in order to produce a model fit with the lowest Akaike information criterion (AIC) possible. Multicollinearity in the final multivariable model, assessed via the variance inflation factor (VIF), was not found to be present. Due to challenges in comparing deprivation across England, Wales, and Scotland a separate multivariable model was produced for England only, with the same methodologies otherwise applied with the addition of IMD decile. All analyses were carried out using R version 4.0.2 (R Core Team, 2020).

## RESULTS

### Data management

When applied to the full SAVSNET database of 6,914,563 consultations, the final regex identified 51,793 veterinary professional‐recorded flea cases (Figure 1) to verify that the case definition (fleas or flea dirt present verified by the attending veterinary practitioner) was satisfied, manual reading of 1000 random records gave a positive predictive value of 98.6%. A different sample of 1000 records was passed to a separate author for manual reading, this process was to verify the success of the extraction method to ensure that the latter results are applicable. Of the 1000 records reviewed, 98 true positives and 896 true negatives were correctly identified by the regex with three false positive and three false negatives were also present. This gives the regular expression a sensitivity of 0.97 and a specificity of 0.99, with precision, recall and F1 also reporting at 0.97. Cohen's Kappa analysis was performed revealing a result of 0.97 suggesting an ‘almost perfect’ agreement (Landis, J. Richard, & Gary G. Koch., 1977). The combination of results from both reviews of the retrieved dataset was deemed sufficiently accurate such that all retrieved cases were included as probable cases in further descriptive analysis and modelling. Cases were excluded if any data entry point was missing, such as age (*N* = 3253), location (*N* = 6964), breed/species (*N* = 5137) or sex (*N* = 1191). In the event, an animal was seen on multiple occasions (regardless of their presence in the case or control cohorts) a record was selected at random, and all others were excluded (*N* = 593). Controls were scrutinized to the same degree, and only used if the data was complete.

### Descriptive findings

**FIGURE 2:**
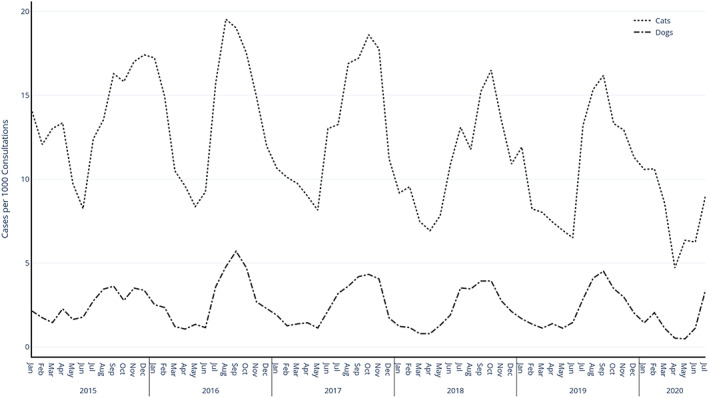
Seasonality plot for cats and dogs as number of recorded flea cases per 1000 consultations from years 2015 to 2020 in Great Britain

Hence, the regex identified fleas as being likely present during 22,276 of 1,902,000 cat consultations (1.17%), and 12,168 of 4,844,850 dog consultations (0.25%). For both dogs and cats, peaks in prevalence between July and August in each year were noted (Figure 2; data here were expressed as a proportion of the total number of recorded cases per 1000 consultations collected by SAVSNET, taking into consideration that these numbers have generally grown annually as the number of practices participating in SAVSNET has grown). Across the sampling period, there was a reduction in the number of cases for each year beyond 2016, particularly observable in cats with 2016 peaking at 19.53 cases per 1000 consultations decreasing to 16.18 in 2019 as represented in Figure 2. Year by year variations in peak months also were seen, with 2015 the highest number of cases was recorded in October, in 2016 in June, followed by August in both 2017 and 2018 and finally July in 2019. Full univariable findings are in Tables S1 and S2.

**FIGURE 3:**
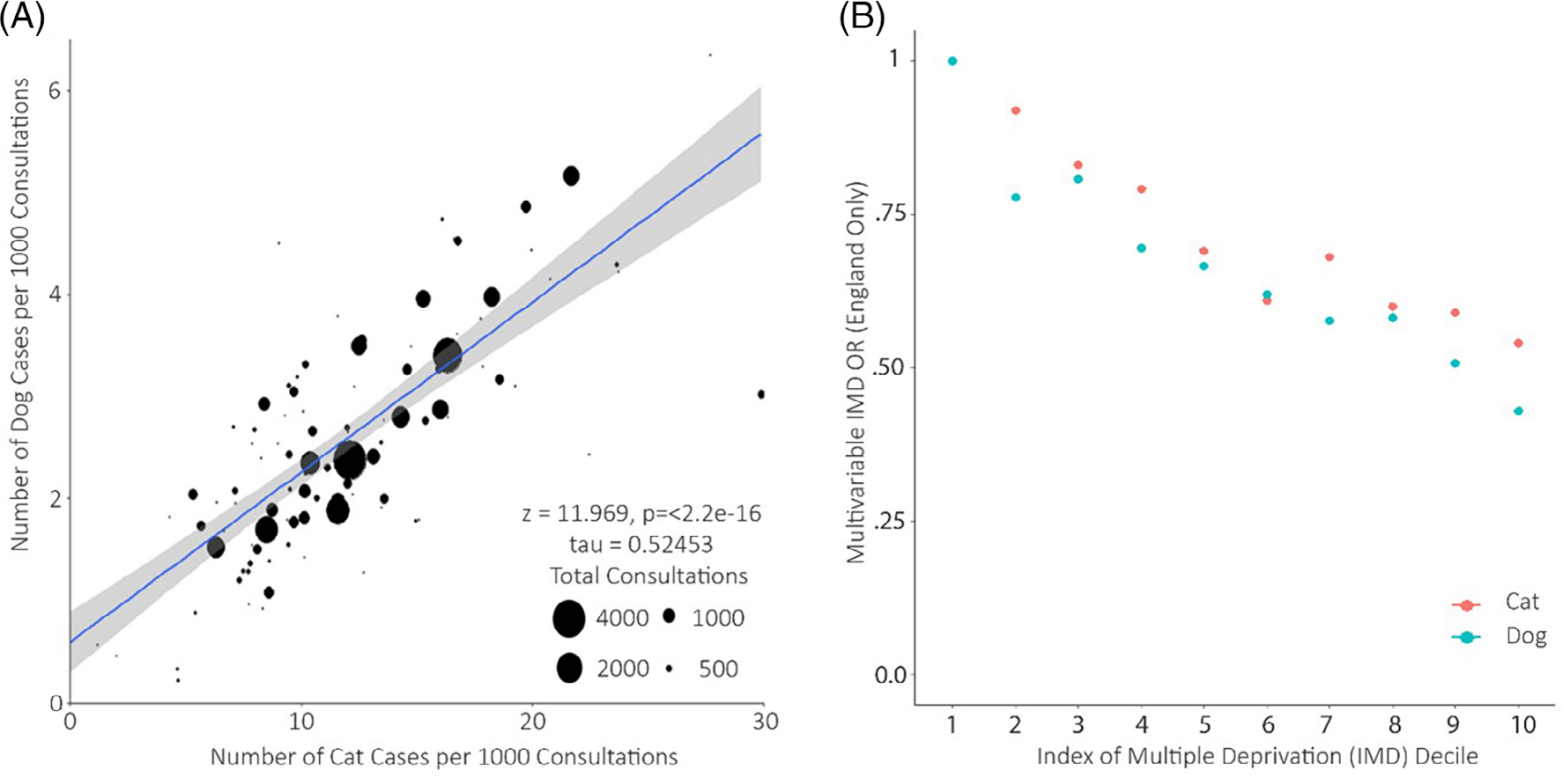
(A) Proportion of flea cases recorded at each practice per 1000 consultations for cats and dogs. Each bubble size is proportional to the total number of consultations contributed by each practice to this study. (B) Odds ratio (OR) of recorded infestation for each index of multiple deprivation (IMD), where the most deprived regions represent the lowest score (1) and the least deprived regions the highest score (10), based on multivariable results from the England ‐only model.

Recorded flea case prevalence in individual practices varied considerably in both cats and dogs; a positive correlation between practice‐level recorded prevalence in dogs and cats was found, suggesting that practices that more commonly recorded flea presence in cats were also likely to record flea presence in dogs (Figure 3; *τ* = 0.52, *z* = 11.97, *p* < 0.001).

### Risk factor modelling

**FIGURE 4:**
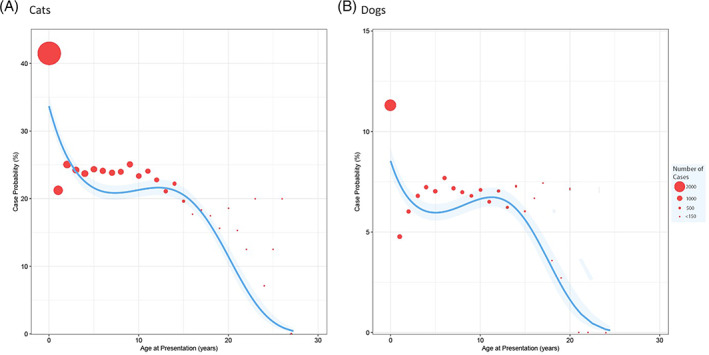
Multivariable analysis for the probability of recorded flea presence against age for both (A) cats and (B) dogs at the time of the consultation, where each bubble size is proportional to the total number of consultations within that category

Multivariable risk factor analysis was performed separately on cats and dogs (Tables 1 and 2). The final model included 22,276 cases for cats and 12,168 for dogs, which were subsequently matched by 255,877 controls. In both species, the odds ratio for neutered animals was approximately half those of entire animals; however, between sexes, there was no significant difference. Crossbreed cats were associated with greatest risk (OR = 1.20, CI = 1.12–1.29, *p* < 0.001), with the Asian breed group being associated with the lowest risk (OR = 0.55, CI = 0.47–0.63, *p* < 0.001). When compared to retrievers, most dog breed groups were associated with increased risk, with the highest risk being in small terriers (OR = 2.83, CI = 2.59–3.08, *p* < 0.001), crossbreed (OR = 2.41, CI = 2.23–2.62, *p* < 0.001) and toy groups (OR = 2.34 CI = 2.13–2.58, *p* < 0.001). Cats and dogs were associated with similar trends in age, showing greatest case probability before the age of 1 before plateauing until age 10 in dogs and 15 in cats (Figure 4).

### Spatial and temporal data

**FIGURE 5:**
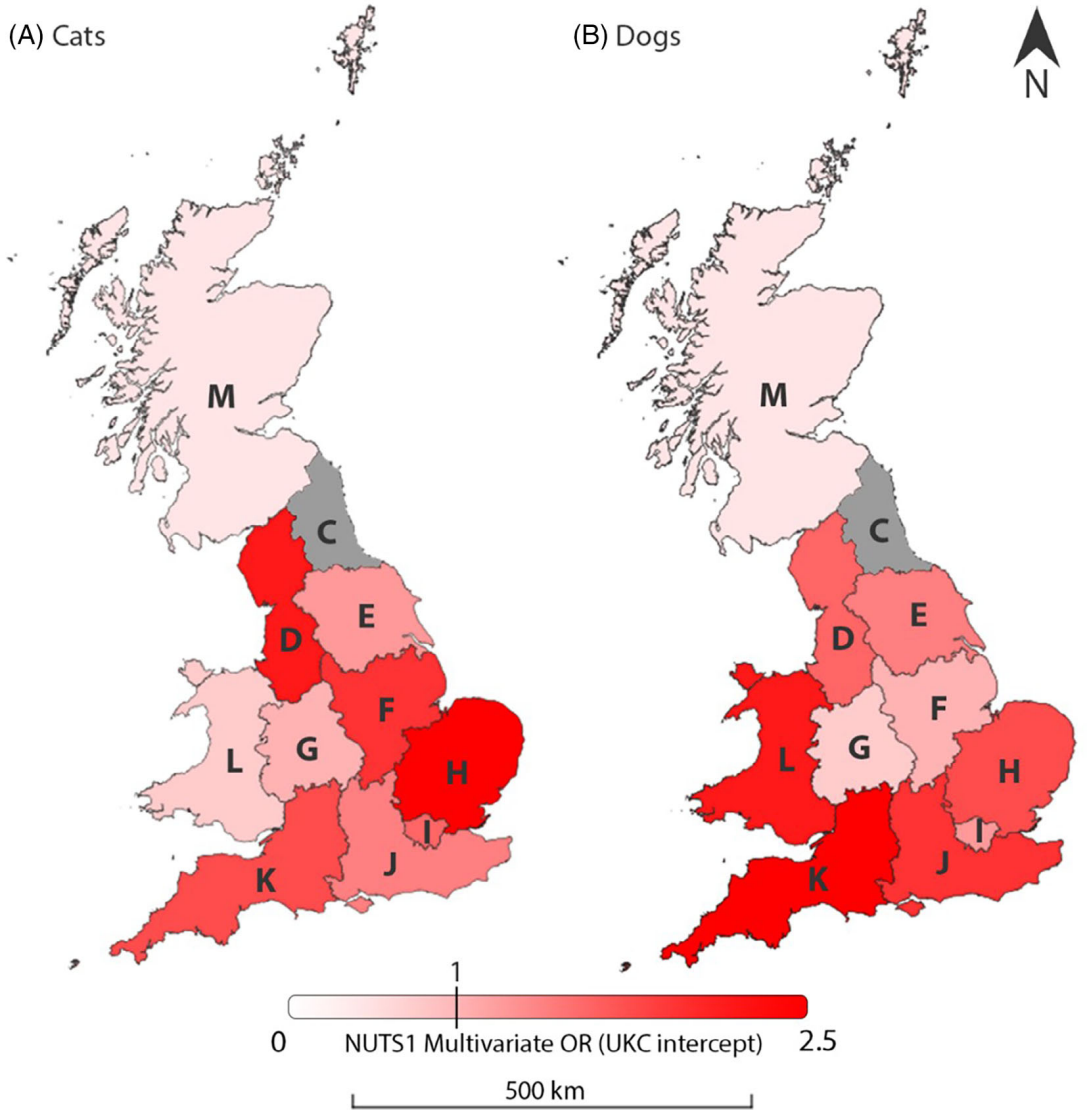
Multivariable odds ratios (OR) for recorded flea presence across Great Britain based on nomenclature of territorial units for statistics (NUTS) 1 regions for both (A) cats and (B) dogs.

When NUTS1 regions were considered within the multivariable model, there was a general trend of reduced recorded cases in more northern NUTS1 regions (Figure 5). For cats, when compared to UKC (North‐East), the greatest odds were observed in UKH (East of England) (OR = 1.53, CI = 1.33–1.76, *p* < 0.001), and in dogs this was UKK (South‐West England) (OR = 2.26, CI = 1.78–2.86, *p* < 0.001). Scotland (UKM) had the lowest odds in both cats and dogs (Cats: 0.34, CI = 0.26–0.43, *p* < 0.001; dogs: OR = 0.76, CI = 0.60–0.97, *p* = 0.025). Multivariable analysis of the month confirmed earlier suggestions of seasonality, when compared to January the lowest odds of recorded infestation being seen was in March in dogs (OR = 0.66, Cl = 0.59–0.75, *p* < 0.001), and May in cats (OR = 0.72, Cl = 0.66–0.78, *p* < 0.001); highest odds were recorded in September for both species (Dogs: 2.56, CI = 2.34–2.80, *p* < 0.001; Cats: OR = 1.40 CI = 1.31–1.50, *p* < 0.001).

As IMD calculation varies between the devolved nations of the UK, thereby limiting comparability between countries, a separate unique multivariable model was constructed for consultations located within England alone and including IMD decile as an additional variable to the finalized model described above. For both dogs and cats, a trend was observed suggesting progressively decreased odds for recorded flea presence among the highest IMD deciles (Figure 3b) (UK Government, 2019). Compared to the lowest deprived decile, the most deprived decile was associated with approximately half the odds (Cats: OR = 0.49, CI = 0.45–0.45, *p* < 0.001; dogs OR = 0.38, CI = 0.34–0.42, *p* < 0.001) of recorded flea presence (Table S3 and S4).

### Other species

Rabbits (*N* = 210) and other species (*N* = 34) consisting of 23 ferrets, five Guinea pigs, two hamsters, two rats, one hedgehog and one cockatiel also were identified as being associated with recorded flea presence in this study. For rabbits, recorded flea cases peaked in September and were lowest in January. Most fleas were recorded on male rabbits, representing 64.06% of cases, and rabbits living in urban environments (70.47%). Age ranged from 0 to 12 years old, with median being 4.04 years. Most cases were seen in the Southeast (UKJ, 22.80%), and the least were found in Scotland and London (UKM/UKI, 0.95%). Due to the small number of cases identified in other species, these were not analysed further here.

## DISCUSSION

Here we have capitalized on the availability of EHRs by analysing data from a six‐year period to give a novel insight into the phenology of fleas associated with companion animals seen at veterinary practices. This we believe provides a pragmatic solution to monitoring flea epidemiology at scale; however, it should be noted that SAVSNET recruits practices based on a convenience sample and as such should not be necessarily representative of the GB pet population as a whole. This might have greatest bearing on risk factors that may be associated with the decision to present the animal to a veterinary practice in the first place, such as IMD.

This considered, consistent with previous practice‐level studies, we have shown that the prevalence of veterinary professional‐recorded flea infestations in cats was approximately twice that of dogs. Although flea species data was not collected, we can speculate that similarities within this study suggest that the cat flea, *C. felis*, is dominant across both host species and aligns with previous flea epidemiology findings (Abdullah et al., 2019; Beck et al., 2006; Dryden & Rust, 1994; Durden et al., 2005). Cats in the UK frequently have unlimited access to the outdoors, and therefore have on‐going exposure to newly emerged fleas, when foraging and hunting. They may frequently contact feral animals and wildlife such as foxes and hedgehogs, which can also serve as hosts for this cosmopolitan ectoparasite (Cooper et al., 2020; Foreman‐Worsley et al., 2021).

The prevalence of infestation reported here for both cats and dogs is much lower than that obtained when animals are purposefully scrutinized for evidence of fleas using a standardized body screening/adult flea sampling protocol in practices recruited for that purpose (Abdullah et al., 2019; Bond et al., 2007). Indeed, these previous UK‐based studies showed that flea infestation exists between 21.09%–28.00% of cats and 6.82%–14.00% of dogs carried fleas. The prevalence of infestation data in this study is therefore not directly comparable due to differences in assessment methodologies, however, our findings demonstrate the utility of continual flea monitoring and suggest that purposeful examination of fleas in UK veterinary practices is rarely occurring.

The effect of temperature and humidity on the cat flea life cycle is well documented (Rothschild, 1975), but how seasonal variation in these parameters affects current phenology in the natural setting is poorly described, owing to the challenges of studying populations longitudinally. Where they are performed, such longitudinal studies are often geographically limited and/or caried out over short time periods. Exploiting the continual annual availability of EHR data, we have shown that flea infestation seasonality is similar for both cats and dogs, and that the greatest number of cases are recorded between July and October in dogs and between July to September in cats, consistent with a previous study in Germany (Beck et al., 2006). Studies carried out on hedgehogs and dogs within Dublin recorded increases in fleas during the summer period of June to August, before dropping off through winter (Baker & Mulcahy, 1986). Interestingly, the peak of recorded cases within our study population varied between years, ranging from August to as late as December in 2015; whether this reflects the temperature and other climatic variations observed within GB is currently unclear. December 2015 recorded the highest average December temperature observed over the study period and may help explain the recorded spike for that year (Meteorological Office UK, 2021). It should however be noted that there is some evidence of seasonal variation in when an animal presents to the veterinary practitioner throughout the year (Singleton et al., 2021), it is possible that this variation could contribute to this observed seasonality.

As well as direct effects on the flea life cycle and increased abundance of fleas, warm weather also influences other behavioural traits such as socializing with increased interactions with other companion animals and generally being outside more. These behavioural factors may explain part of the high number of recorded cases observed at certain times of the year (Foreman‐Worsley et al., 2021; Hamilton et al., 2008). On a year‐by‐year basis, the number of recorded flea cases per 1000 consultations within our dataset appeared to be declining despite an increase in the number of practices participating in SAVSNET. This trend may indicate that new types of flea treatments are becoming more effective, as the year‐on‐year decline coincides with the introduction of drugs in the isoxazoline class of ectoparasiticide in 2015. Studies in this class have shown very high efficacies against *C. felis* within companion animals (Kunkle et al., 2014). This observation requires further research and monitoring in future years since understanding such patterns and how they are affected by prophylactic treatments will help inform future therapeutic interventions (Dryden & Rust, 1994). It should be noted that the observed drop off in observed cases in April 2020 coincides with GB entering COVID‐19 lockdown restrictions and therefore may not be due to changes in flea abundance but rather a reduction in overall consultations.

Consistent with the only other previous study on geographical distribution in the UK, cases were seen to fluctuate across the country, with an overall trend of decreasing numbers as latitudes increased, such northerly latitudes being generally associated with cooler temperatures (Cooper et al., 2020). However, flea cases were recorded in every month and in every year, regardless of region; this may be explained by flea survival in the environment during low temperatures with induced quiescence in colder weather but with periods of activity during warmer spells. Continuous development of the immature flea stages in warm houses during colder weather is also known to occur (Carlotti & Jacobs, 2000).

There was an observed association with increased risk of fleas with breed, with small terriers and toy dogs being at greater risk compared to the retriever breed group presenting within the clinical notes. Crossbreeds had increased odds in both cats and dogs, and in cats in particular were observed to be at greatest risk. To our knowledge, this is the first study to suggest different breed types may have differing risks. Possible reasons for this observation are that small dogs, like cats, are in a closer physical proximity to vegetation and animals' burrows or resting places where newly emerged fleas may be present, thus proving a more opportunistic flea encounter through stronger cues for flea attraction. However, it is also possible that the smaller size of these animals may increase a veterinary practitioner's opportunity to discover evidence of fleas or flea dirt. Length of fur and indeed the colour of the coat may also affect the chance of flea observations, with longer hair possibly providing on the one hand increased surface area for attachment and on the other, greater opportunity for fleas to remain unobserved (and therefore unrecorded). This study did not record details on the coat length or type, and therefore was not analysed here. Furthermore, behavioural differences observed in different breeds or indeed varying behaviour in owners that choose to own different breeds may all contribute to factors increasing or decreasing odds of flea infestation.

Age of the pet produced similar trends of recorded flea risk in both cats and dogs, with the highest risk of infestation being seen in animals aged up to 1‐year‐old. Possible reasons are wide ranging and speculative and include greater attraction of fleas towards younger animals, older animals possibly venturing outside and interacting less, more time spent examining young animals during their first presentations at practice, or the small size of younger animals increasing the chance of noticing fleas (Reeves et al., 2011; Roul et al., 2011). In addition, older dogs visiting the veterinary practice may be more likely to be attending for a pre‐existing medical condition, leading the attending veterinary professional to focus on this issue in preference to looking for, or recording, fleas. Interestingly, findings of a study of the flea *Siphonaptera Pulicidae Xenopsylla ramesis* (Rothschild, 1904) feeding on the rodent *Rodentia Muridae Meriones crassus* (Sundevall, 1842) showed that the size of a flea blood meal was largest when fleas fed on juveniles, and, by extension, the lifespan was seen to be longest in these fleas, possibly alluding to a longer persistence on and preference for juvenile animals (Roul et al., 2011). Although the models constructed here broadly capture age‐based variability in flea infestation risk, rapid variation in case probability was noted particularly between animal ages 0–1, and 1–2 years of age in both cats and dogs. For these ages our modelling approach may have under and overestimated case probability, respectively. In future work, we aim to develop methods by which this interesting finding may be modelled more precisely.

In both cats and dogs, neutered animals were associated with reduced risk compared to those that were non‐neutered (entire). Although biological and behavioural factors might explain this finding, owners residing within more deprived areas have previously been shown to be less engaged with preventive veterinary care (Sánchez‐Vizcaíno et al., 2018). Thus our finding here might be more suggestive of differences in owner engagement with preventive veterinary care (i.e., the decision to provide antiparasitic treatment less frequently to their animal) than there being a direct biological association between neuter status and flea infestation risk. Interestingly, we also observed that animals whose owners originated from more deprived areas of England were also associated with increased odds of recorded flea infestation. This does suggest that the ability to pay for flea control is having an impact on the odds of recorded flea infestations. Although flea treatment is readily and comparatively cheaply available in most supermarkets, those generally recommended by veterinary professionals as being more effective are only available via a prescription, and are likely to be associated with greater costs for the owner. It is possible that we are observing this inequity in our findings here, though we would like to remind the reader of the potential limitations associated with the convenience sampling used in our study. This considered, our findings suggest a potential avenue for targeted healthcare measures towards higher deprivation areas as a means to reduce flea prevalence, whether this be in reducing flea treatment costs or increasing availability of information.

Data from EHRs provides a rich source of practical information but does come with certain limitations. Although significant efforts were made to capture as many cases as possible here, it is inevitable that some were missed, either because our regular expression methodology did not identify the specific expression used in participating veterinary practices, or simply that the presence of fleas or flea dirt seen during a given consultation was not recorded. In addition, since our regex is not 100% accurate, a small proportion of false positive cases are included in the analysis. Another limitation is that veterinary practitioners rarely attempt to identify flea species infestation. Other potential study criticisms are that the data is restricted to those presenting at a SAVSNET participating practice, where recruitment is largely based on convenience, such that the findings of this study cannot be considered representative of the entire population of vet‐visiting companion animals in GB. National coverage of SAVSNET data is variable such that accuracy of estimates will vary, as SAVSNET grows it is hoped that some of these coverage issues will be addressed.

To conclude, this study has used an extensive novel dataset and text mining methodology to analyse large volumes of EHR data assimilated over a six‐year period. We described the seasonality, geographical distribution and risk factors associated with recorded flea cases across GB. These newly identified factors such as variances in breed types, age, and neuter status to name a few, could form the basis of more targeted health messages for veterinary practitioners and companion animal owners, allowing prophylactic and therapeutic treatments to be used on those animals most at risk, and at the right time. This might ultimately reduce empirical flea treatment use, which has recently been shown to be contaminating rivers having great environmental impacts on aquatic life (Perkins et al., 2021). The method we describe here can provide a real‐time, cost‐effective contribution to monitoring strategy as a foundation for ongoing flea surveillance in small animals.

## AUTHOR CONTRIBUTIONS

Alan D. Radford, John McGarry, David A. Singleton and Sean Farrell conceived the study. Sophie Cantwell and Sean Farrell completed preliminary case identification work; Sean Farrell extended and applied this work into the main dataset supervised by P. J.‐M. Noble, Alan D. Radford, Gina J. Pinchbeck and David A. Singleton devised and completed statistical analysis. Sean Farrell drafted the manuscript which was reviewed by all authors.

## CONFLICT OF INTEREST

David A. Singleton, Alan D. Radford, PJ‐M Noble, Gina J Pinchbeck and John McGarry have previously received funding from pharmaceutical companies that produce ectoparasiticides. The remaining authors declare no conflicts of interest.

## Supporting information


**Data S1.** Supporting information.

## Data Availability

Data is available by reasonable request to SF.
