# Supplementary material for: Seasonality and other risk factors for fleas infestations in domestic dogs and cats
Source: Med Vet Entomol. 2023 Jan 9;37(2):359–70. doi: 10.1111/mve.12636 (PMC10946788; doi:10.1111/mve.12636)
Supplement: Supplementary file 1 — Data S1. Supporting information. [file MVE-37-359-s001.docx]

**Python Regular Expression (regex) for the extraction of suspected flea cases from the SAVSNET EHR dataset free text narratives**

(fle[ea]s?\s?)(comb\s?|brush\s?)(\+|\+ve[3]|ve\+|positive|passed|\+)|(\+|\+ve[3]|ve\+[3]|positive|passed|\+)\s(fle[ea]s?\s)(comb\s?|brush\s?)|(wet paper|wpt)(\sve+|\s\+ve|ve\+|\+ve|\s\+|\+|positive|passed|\+)|(\+|\+ve|\bve\+\b|positive|passed|\+)\s(wet paper (test)?|wpt)|(?<!no\s)fleas?\s?in\s?(comb|brush)|fle[ea]s?\stests?\s(\+|\+ve[3]|ve\+|pos|pass|\+)|(?<!\/)(?<!\/\s)(?<!any\s)(?<![o|w|R|\'s|\']\s)(?<!not\s)(?<!no visible\s)(?<!advice\s)(?<!n\'t\s)(?<!no immediate\s)(?<!no\s)(?<!Check\s)(?<![o|w|R|\'|\']\sreports\s)(?<![o|w|R|\']\sdid see\s)(?<![o|w|R|\']\sreported\s)(?<!reports\s)(?<!report\s)(?<![o|w|R|\']\s)(?<!did\s)(?<!said\s)(?<!may have\s)(?<!can\s)(?<!may\s)(?<!o find\s)(?<!booster\s)(?<!she\s)(?<!\bas\b)(?<!he\s)(?<!treated\s)(?<!If still\s)(?<!maybe\s)(?<!was\s)(?<!concerned\s)(?<!might\s)(?<!suspects\s)(?<!bitten by\s)(?<!thinks\s)(?<!cat\s)(?<![o|w|R|\'] ha[ds]\s)(?<!vous\s)(?<!concerned about\s)(?<!Never\s)(?<!when sees\s)(?<![o|w|R|\'] noticed a\s)(?<![o|w|R|\'] says\s)(?<!has\s)(?<![o|w|R|\'|ers]\ssaw\s)(?<!kc\s)(?<![o|w|R|\'|s]ha[vds]\sseen\s)(?<![o|w|R|\']\saware\s)(?<!Weigh\s)(?<![o|w|R|\']\sseen\s)(?<![o|w|R|\']\sstill\s)(?<!had\s)(?<!advised\s)(?<!recently\s)(?<!felv\s)(?<!t see\s)(?<!t\s)(?<!possibly\s)(?<!if\s)(?<!poss\s)(?<!possible\s)(?<![o|w|R|\'] noticed a\s)(?<![o|w|R|\'] found\s)(?<![o|w|R|\'] finding\s)(?<![o|w|R|\'] has found\s)(?<![o|w|R|\']\snoticed\s)(?<![o|w|R|\']\sha[ds]\snoticed\s)(?<![o|w|R|\'|s]\shave\s)(?<![o|w|R|\']\sha[ds]\sseen\s)(?<!think\s)(?<!worm\s)(?<!vacc\s)(?<!vaccs\s)(?<!wormers\s)(?<!worm)(?<!worming\s)(?<!worming)(?<!cR)(?<!wormer)(?<!wormer\s)(?<!disc\s)(?<!discuss\s)(?<!reports\s)(?<!discussed\s)(?<!utd\s)(?<!date\s)(?<!no obvious\s)(?<![o|w|R|\'] ha[ds]\s)(?<!not\s)(?<!tx\s)(ve+[3]|\+ve|positive|\+\+|\d|\d\s?x|saw( |\sa)|has( |\sa)|have|some|saw|seeing( |\sa)|moderate|multiple|minimal|severe|big|massive|infested (in|with)|signs of|hooching (in|with)|infestation of|few|but|finding|loads of|lots? of|\bone\b|two|three|amount of|evidence of|mild|apart from|except|seen( |\sa)|noticed( |\sa)|found( |\sa)|single|plenty of|obvious|couple( |\sof)obv|many|in{2}|riddled\s(in|with)|issues with\s|covered (in|with))(\sa?live|\sliving|\sadult)?\s?(\bfle[ea]s?\b)(?!(\'d|ed|d|\sworm|\stick))(?!(\sneg|\bve-\b|\bve-\b))(?!(\+|./|\s\+|\s\?|\\|\/|\s?/|\sand|\sor|and|\s?&|or)\s?(worm|mite|worming|tick|w|de|par))(?!\s?tre?|\s?pow|\s?bo|\s?tr?x|\s?col|\s?all|\s?sp|\s?com|\s?con|\stab|\sprod|\sbite|\spre|\sder|\srep)|(?<![o|w|R|\']\s)(?<!\/)(?<!\/\s)(?<!any\s)(?<!not\s)(?<!advice\s)(?<!n\'t\s)(?<!no\s)(?<!no\slive\s)(?<!no lives\s)(?<!no\sliving\s)(?<!reports\s)(?<!report\s)(?<![o|w|R|\']\s)(?<!o\snoticed)(?<!did\s)(?<!may\s)(?<!booster\s)(?<!she\s)(?<!he\s)(?<!treated\s)(?<!maybe\s)(?<!might\s)(?<!suspects\s)(?<!thinks\s)(?<!think\s)(?<![o|w|R|\'] ha[ds]\s)(?<!has\s)(?<!advised\s)(?<!possibly\s)(?<!poss\s)(?<![o|w|R|\'] noticed\s)(?<!possible\s)(?<!no obvious\s)(?<!worm\s)(?<!No flea dirt[\sor\s|\/|\s\/|\s\/\s]\s)(?<!worming\s)(?<!disc\s)(?<!discuss\s)(?<!reports\s)(?<!No more\s)(?<!discussed\s)(?<!no signs of\s)(?<!no sign of\s)(?<!no evidence of\s)(?<!utd\s)(?<!date\s)(?<![o|w|R|\'] ha[ds]\s)(?<!not\s)(?<!tx\s)(living\s|a?live\s|adult\s)?(\bfle[ea]s?\b)\s?(!|\bve+\b|\b\+ve\b|\bve+\b|\b\+ve\b|\+|\s\+|\+\s|noted|positive|evidence of|found|detect|severe|noticed|crawling\s(with|in)|saw|in consult|lots of|loads|present|infest|every\s?where|caught|dropped)(?!\s?worm)(?!\s?tick)(?!\sby [o|w|R|\']|\sat home|worming)(?!(\+|\s\+|\+\s|\s\?|\|/|\s?/|\sand|\sor|and|\s?&|or)\s?(worm|mite|worming|tick|w|r))(?!\s?tre?|\s?tr?x|\s?bo|\s?col|\s?sp|\s?com|\s?con|w|\s?tab|ing)|(?<!/\s)(?<!/)(?<!no\s)(?<!not\s)(?<![o|w|R|\'] has\s)(?<![o|w|R|\'] found\s)(?<!No flea dirt[\sor\s|\/|\s\/|\s\/\s]\s)(?<!o noticed\s)(?<!any)(ve+[3]|\+ve|positive|\+|\d|has|have|seen|saw|some|infested with|infestation of|loads of|lots of|evidence of|found|live|plenty of|except|many|in{2}|riddled in|covered in\s)(fle[ae]s?\s?dirts?)|(?<!\/\s)(?<!/)(?<!or\s)(?<!no\s)(?<!not\s)(?<!no obvious\s)(obvious\s|clear\s|but\s|evidence of\s|\.\s)?(fle[ae]s?\s?dirt[ys]?)(\sve+|ve+|\s\+ve|\+ve|\+|\s?\+|\spos|\sevid|\sfound|\sin|\sdetect|\scrawl|\spresent|\sspotted|\sinfest|\scover|\sseen|\severy|\scaught|\sdropped|\sinfest)|(?<!no\s)(live|dead|alive|living|adult|evidence of)\s(fle[ea]s?\s?(seen))|\.\s?fle[ae]s?\.

# Supplementary Table 1 – Risk of veterinary-recorded flea infestation in dogs (N=12,168) from Univariable logistic regression model throughout Great Britain. ^a^ Standard Error, ^b^ Odds Ratio and ^c^ 95% Confidence Interval

| Variable | Level | Case | Controls | beta | se | OR (95% CI) | P |
| --- | --- | --- | --- | --- | --- | --- | --- |
| **Categorical Variables** | | | | | | | |
| Sex | Male (intercept) | 6385 (52.5) | 77214 (48.9) | -2.635 | 0.036 | 1.00 | - |
|  | Female | 5781 (47.5) | 80689 (51.1) | -0.05 | 0.019 | 0.95 (0.92-0.99) | 0.008 |
| Neutered | Neutered (intercept) | 6139 (50.5) | 56994 (36.1) | -2.925 | 0.037 | 1.00 | - |
|  | Entire | 6029 (49.5) | 100909 (63.9) | 0.618 | 0.019 | 1.86 (1.79-1.93) | <0.001 |
| Sex + Neuter Status | Male Entire (intercept) | 3264 (26.8) | 31029 (19.7) | -2.334 | 0.039 | 1.00 | - |
|  | Male Neutered | 3122 (25.7) | 49660 (31.4) | -0.543 | 0.026 | 0.58 (0.55-0.61) | <0.001 |
|  | Female Entire | 2875 (23.6) | 25965 (16.4) | 0.058 | 0.027 | 1.06 (1.00-1.12) | 0.034 |
|  | Female Neutered | 2907 (23.9) | 51249 (32.5) | -0.64 | 0.027 | 0.53 (0.50-0.56) | <0.001 |
| Urban | Rural (intercept) | 3515 (28.9) | 51184 (32.4) | -2.773 | 0.039 | 0.06 (0.06-0.07) | <0.001 |
|  | Urban | 8653 (71.1) | 106719 (67.6) | 0.17 | 0.025 | 1.19 (1.13-1.25) | <0.001 |
| NUTS1 | UKC (North East - intercept) | 704 (5.8) | 14337 (9.1) | -3.027 | 0.082 | 1.00 | - |
|  | UKD (North West) | 1328 (10.9) | 16167 (10.2) | 0.469 | 0.108 | 1.60 (1.29-1.98) | <0.001 |
|  | UKE (Yorks and Humber) | 1157 (9.5) | 17843 (11.3) | 0.357 | 0.109 | 1.43 (1.16-1.77) | 0.001 |
|  | UKF (East Midlands) | 637 (5.2) | 9797 (6.2) | 0.119 | 0.116 | 1.13 (0.90-1.41) | 0.304 |
|  | UKG (West Midlands) | 699 (5.7) | 11937 (7.6) | 0.094 | 0.125 | 1.10 (0.86-1.40) | 0.451 |
|  | UKH (East of England) | 1691 (13.9) | 19295 (12.2) | 0.448 | 0.114 | 1.57 (1.25-1.96) | <0.001 |
|  | UKI (Greater London) | 110 (0.9) | 1708 (1.1) | 0.297 | 0.146 | 1.35 (1.01-1.79) | 0.042 |
|  | UKJ (South East) | 2763 (22.7) | 32446 (20.5) | 0.5 | 0.098 | 1.65 (1.36-2.01) | <0.001 |
|  | UKK (South West) | 1891 (15.5) | 18307 (11.6) | 0.703 | 0.109 | 2.02 (1.63-2.50) | <0.001 |
|  | UKL (Wales) | 885 (7.3) | 7913 (5) | 0.679 | 0.131 | 1.97 (1.53-2.55) | <0.001 |
|  | UKM (Scotland) | 302 (2.5) | 8153 (5.2) | 0.353 | 0.112 | 0.703 (0.57-0.88) | 0.002 |
| Months | January (intercept) | 726 (6) | 13927 (8.8) | -3.038 | 0.05 | 1.00 | - |
|  | February | 650 (5.3) | 13353 (8.5) | -0.062 | 0.055 | 0.94 (0.84-1.05) | 0.254 |
|  | March | 453 (3.7) | 13125 (8.3) | -0.416 | 0.06 | 0.66 (0.59-0.74) | <0.001 |
|  | April | 418 (3.4) | 11122 (7) | -0.331 | 0.062 | 0.72 (0.64-0.81) | <0.001 |
|  | May | 439 (3.6) | 11631 (7.4) | -0.336 | 0.061 | 0.71 (0.63-0.81) | <0.001 |
|  | June | 656 (5.4) | 12764 (8.1) | -0.029 | 0.055 | 0.97 (0.87-1.08) | 0.596 |
|  | July | 1406 (11.6) | 14963 (9.5) | 0.575 | 0.047 | 1.78 (1.62-1.95) | <0.001 |
|  | August | 1698 (14) | 13970 (8.8) | 0.833 | 0.045 | 2.30 (2.11-2.52) | <0.001 |
|  | September | 1846 (15.2) | 13638 (8.6) | 0.938 | 0.045 | 2.56 (2.34-2.79) | <0.001 |
|  | October | 1708 (14) | 14145 (9) | 0.825 | 0.045 | 2.28 (2.09-2.49) | <0.001 |
|  | November | 1288 (10.6) | 13061 (8.3) | 0.629 | 0.047 | 1.88 (1.71-2.058) | <0.001 |
|  | December | 879 (7.2) | 12204 (7.7) | 0.316 | 0.051 | 1.37 (1.24-1.52) | <0.001 |
| Breeds | Retriever (intercept) | 122 (1) | 2041 (1.3) | -3.276 | 0.049 | 1.00 | - |
|  | Ancient Spitz | 3322 (27.3) | 33811 (21.4) | 0.387 | 0.099 | 1.47 (1.21-1.79) | <0.001 |
|  | Crossbreed | 619 (5.1) | 6823 (4.3) | 0.852 | 0.04 | 2.35 (2.17-2.54) | <0.001 |
|  | Herding | 642 (5.3) | 14699 (9.3) | 0.79 | 0.055 | 2.20 (1.98-2.45) | <0.001 |
|  | Mastiff Like | 789 (6.5) | 19339 (12.2) | 0.053 | 0.054 | 1.06 (0.95-1.17) | 0.319 |
|  | Scent Hound | 178 (1.5) | 3889 (2.5) | 0.084 | 0.084 | 1.09 (0.92-1.28) | 0.315 |
|  | Sight Hound | 112 (0.9) | 2456 (1.6) | 0.088 | 0.102 | 1.09 (0.90-1.33) | 0.385 |
|  | Small Terrier | 1967 (16.2) | 17092 (10.8) | 1.004 | 0.043 | 2.73 (2.51-2.97) | <0.001 |
|  | Spaniel | 1464 (12) | 18705 (11.8) | 0.642 | 0.045 | 1.90 (1.74-2.085) | <0.001 |
|  | Toy | 1132 (9.3) | 11150 (7.1) | 0.899 | 0.047 | 2.46 (2.24-2.70) | <0.001 |
|  | Unclassified | 1462 (12) | 21774 (13.8) | 0.504 | 0.045 | 1.66 (1.52-1.81) | <0.001 |
|  | Working Dog | 358 (2.9) | 6124 (3.9) | 0.358 | 0.065 | 1.43 (1.26-1.62) | <0.001 |
| **Continuous Variables** | | | | | | | |
| Age |  |  |  | 0.014 | 0.011 | 0.99 (0.97-1.01) | 0.192 |

**Supplementary table 2 – Risk of veterinary-recorded flea infestation in cats (N=22,276) from Univariable logistic regression model throughout Great Britain throughout Great Britain. ^a^ Standard Error, ^b^ Odds Ratio and ^c^ 95% Confidence Interval**

| **Variable** | **Level** | **Case (%)** | **Control (%)** | **Beta** | **SE** | **OR (95% CI)** | **P** |
| --- | --- | --- | --- | --- | --- | --- | --- |
| Sex | Male (intercept) | 10889 (48.9) | 32522 (48.5) | -1.189 | 0.032 | 1.00 | - |
|  | Female | 11387 (51.1) | 34504 (51.5) | -0.012 | 0.016 | 0.988 (0.958-1.019) | 0.457 |
| Neutered | Neutered (intercept) | 6365 (28.6) | 11952 (17.8) | -1.343 | 0.032 | 1.00 | - |
|  | Entire | 15911 (71.4) | 55074 (82.2) | 0.627 | 0.018 | 1.872 (1.806-1.941) | <0.001 |
| Sex + Neuter Status | Male Entire (intercept) | 2995 (4.9) | 5431 (6) | -0.679 | 0.038 | 1.00 | - |
|  | Male Neutered | 15788 (25.9) | 54182 (60) | -0.658 | 0.027 | 0.518 (0.491-0.545) | <0.001 |
|  | Female Entire | 10110 (16.6) | 19563 (21.6) | -0.07 | 0.032 | 0.932 (0.876-0.992) | 0.027 |
|  | Female Neutered | 32068 (52.6) | 11193 (12.4) | -0.671 | 0.027 | 0.511 (0.485-0.539) | <0.001 |
| Urban | Rural (intercept) | 5033 (22.6) | 17765 (26.5) | -1.337 | 0.035 | 1.00 | - |
|  | Urban | 17243 (77.4) | 49261 (73.5) | 0.204 | 0.023 | 1.226 (1.173-1.281) | <0.001 |
| NUTS1 | UKC (North East - intercept) | 821 (3.7) | 3454 (5.2) | -1.346 | 0.103 | 1.00 | - |
|  | UKD (North West) | 2425 (10.9) | 6421 (9.6) | 0.242 | 0.126 | 1.274 (0.994-1.632) | 0.055 |
|  | UKE (Yorks and Humber) | 2077 (9.3) | 6527 (9.7) | 0.04 | 0.132 | 1.041 (0.805-1.347) | 0.758 |
|  | UKF (East Midlands) | 1195 (5.4) | 4049 (6) | 0.018 | 0.133 | 1.018 (0.784-1.32) | 0.895 |
|  | UKG (West Midlands) | 1530 (6.9) | 4589 (6.8) | 0.098 | 0.137 | 1.102 (0.843-1.443) | 0.477 |
|  | UKH (East of England) | 3055 (13.7) | 8743 (13) | 0.091 | 0.13 | 1.095 (0.849-1.414) | 0.484 |
|  | UKI (Greater London) | 402 (1.8) | 991 (1.5) | 0.314 | 0.162 | 1.369 (0.997-1.88) | 0.052 |
|  | UKJ (South East) | 5924 (26.6) | 18497 (27.6) | 0.239 | 0.116 | 1.269 (1.011-1.593) | 0.04 |
|  | UKK (South West) | 3233 (14.5) | 8035 (12) | 0.41 | 0.127 | 1.507 (1.175-1.934) | 0.001 |
|  | UKL (Wales) | 1079 (4.8) | 2908 (4.3) | 0.272 | 0.154 | 1.313 (0.971-1.775) | 0.077 |
|  | UKM (Scotland) | 535 (2.4) | 2812 (4.2) | -0.45 | 0.126 | 0.637 (0.498-0.815) | <0.001 |
| Months | January (intercept) | 1862 (1.2) | 5983 (1.3) | -1.254 | 0.04 | 1.00 | - |
|  | February | 3028 (1.9) | 11224 (2.5) | -0.14 | 0.04 | 0.869 (0.804-0.939) | <0.001 |
|  | March | 3723 (2.3) | 15921 (3.6) | -0.281 | 0.042 | 0.755 (0.696-0.819) | <0.001 |
|  | April | 3920 (2.4) | 17824 (4) | -0.347 | 0.045 | 0.706 (0.647-0.771) | <0.001 |
|  | May | 4950 (3.1) | 23480 (5.3) | -0.415 | 0.044 | 0.66 (0.605-0.72) | <0.001 |
|  | June | 7950 (4.9) | 31332 (7.1) | -0.207 | 0.041 | 0.813 (0.75-0.881) | <0.001 |
|  | July | 15337 (9.5) | 43043 (9.7) | 0.128 | 0.037 | 1.136 (1.057-1.221) | 0.001 |
|  | August | 19536 (12.1) | 47616 (10.7) | 0.268 | 0.036 | 1.307 (1.217-1.403) | <0.001 |
|  | September | 23526 (14.6) | 52452 (11.8) | 0.36 | 0.036 | 1.433 (1.335-1.537) | <0.001 |
|  | October | 28260 (17.5) | 64480 (14.5) | 0.332 | 0.035 | 1.393 (1.3-1.493) | <0.001 |
|  | November | 27291 (16.9) | 65824 (14.8) | 0.282 | 0.036 | 1.326 (1.235-1.423) | <0.001 |
|  | December | 21720 (13.5) | 64668 (14.6) | 0.081 | 0.038 | 1.084 (1.005-1.169) | 0.036 |
| Breeds | West Europe (intercept) | 1075 (4.8) | 3656 (5.5) | -1.309 | 0.046 | 1.00 | - |
|  | Asian | 244 (1.1) | 1743 (2.6) | -0.744 | 0.077 | 0.475 (0.409-0.552) | <0.001 |
|  | Crossbreed | 20478 (91.9) | 59683 (89) | 0.147 | 0.036 | 1.158 (1.079-1.243) | <0.001 |
|  | Mediterranean | 6 (0.03) | 24 (0.04) | -0.221 | 0.452 | 0.802 (0.331-1.943) | 0.624 |
|  | Unclassified | 473 (2.1) | 1920 (2.9) | -0.188 | 0.063 | 0.829 (0.733-0.938) | 0.003 |
| Age |  | - | - |  |  |  |  |

#

# Supplementary Table 3 – Risk of veterinary-recorded flea infestation in dogs (N=12,168) for England Only with IMD . ^a^ Standard Error, ^b^ Odds Ratio and ^c^ 95% Confidence Interval

| **Variable** | **Level** | **Case (%)** | **Controls (%)** | **beta** | **SE^a^** | **OR^B^ (95% CI^C^)** | **P** |
| --- | --- | --- | --- | --- | --- | --- | --- |
|  | (Intercept) |  |  | -3.843 | 0.113 | - | - |
| **Categorical Variables** | | | | | | | |
| Sex + Neuter | Male Entire | 3264 (26.8) | 31029 (19.7) | - | - | 1.00 | - |
|  | Male Neutered | 3122 (25.7) | 49660 (31.4) | -0.061 | 0.059 | 0.58 (0.55 - 0.61) | <0.001 |
|  | Female Entire | 2875 (23.6) | 25965 (16.4) | -0.437 | 0.065 | 1.06 (1 - 1.12) | 0.064 |
|  | Female Neutered | 2906 (23.9) | 51249 (32.5) | -0.316 | 0.066 | 0.54 (0.51 - 0.57) | <0.001 |
| Months | January | 726 (6) | 13927 (8.8) | - | - | - | - |
|  | February | 650 (5.3) | 13353 (8.5) | -0.061 | 0.059 | 0.94 (0.84 - 1.06) | 0.298 |
|  | March | 453 (3.7) | 13125 (8.3) | -0.437 | 0.065 | 0.65 (0.57 - 0.73) | <0.001 |
|  | April | 418 (3.4) | 11122 (7) | -0.316 | 0.066 | 0.73 (0.64 - 0.83) | <0.001 |
|  | May | 439 (3.6) | 11631 (7.4) | -0.347 | 0.066 | 0.71 (0.62 - 0.8) | <0.001 |
|  | June | 656 (5.4) | 12764 (8.1) | -0.041 | 0.059 | 0.96 (0.86 - 1.08) | 0.482 |
|  | July | 1406 (11.6) | 14963 (9.5) | 0.578 | 0.05 | 1.78 (1.62 - 1.97) | <0.001 |
|  | August | 1698 (14) | 13970 (8.8) | 0.81 | 0.049 | 2.25 (2.04 - 2.48) | <0.001 |
|  | September | 1846 (15.2) | 13638 (8.6) | 0.941 | 0.048 | 2.56 (2.33 - 2.82) | <0.001 |
|  | October | 1708 (14) | 14145 (9) | 0.836 | 0.049 | 2.31 (2.1 - 2.54) | <0.001 |
|  | November | 1288 (10.6) | 13061 (8.3) | 0.633 | 0.051 | 1.88 (1.7 - 2.08) | <0.001 |
|  | December | 879 (7.2) | 12204 (7.7) | 0.32 | 0.055 | 1.38 (1.24 - 1.53) | <0.001 |
| Rural/Urban | Rural | 3515 (28.9) | 51184 (32.4) | - | - | 1.00 | - |
|  | Urban | 8653 (71.1) | 106719 (67.6) | 0.09 | 0.028 | 1.09 (1.04 - 1.16) | <0.001 |
| NUTS1 | **UKC** (North East) | 704 (5.8) | 14337 (9.1) |  |  | 1.00 | - |
|  | UKD (North West) | 1328 (10.9) | 16167 (10.2) | 0.571 | 0.119 | 1.77 (1.4 - 2.24) | <0.001 |
|  | UKE (Yorks & Humber) | 1157 (9.5) | 17843 (11.3) | 0.477 | 0.123 | 1.61 (1.27 - 2.05) | <0.001 |
|  | UKF (East Midlands) | 637 (5.2) | 9797 (6.2) | 0.31 | 0.129 | 1.36 (1.06 - 1.75) | 0.016 |
|  | UKG (West Midlands) | 699 (5.7) | 11937 (7.6) | 0.167 | 0.132 | 1.18 (0.91 - 1.53) | 0.207 |
|  | UKH (East of England) | 1691 (13.9) | 19295 (12.2) | 0.651 | 0.122 | 1.92 (1.51 - 2.44) | <0.001 |
|  | UKI (Greater London) | 110 (0.9) | 1708 (1.1) | 0.433 | 0.168 | 1.54 (1.11 - 2.14) | 0.01 |
|  | UKJ (South East) | 2763 (22.7) | 32446 (20.5) | 0.752 | 0.112 | 2.12 (1.7 - 2.64) | <0.001 |
|  | UKK (South West) | 1891 (15.5) | 18307 (11.6) | 0.882 | 0.12 | 2.42 (1.91 - 3.06) | <0.001 |
| Breed | Retriever | 122 (1) | 2041 (1.3) |  |  | 1.00 | - |
|  | Ancient Spitz | 3322 (27.3) | 33811 (21.4) | 0.332 | 0.107 | 1.39 (1.13 - 1.72) | 0.002 |
|  | Crossbreed | 619 (5.1) | 6823 (4.3) | 0.876 | 0.044 | 2.40 (2.20 - 2.62) | <0.001 |
|  | Herding | 642 (5.3) | 14699 (9.3) | 0.819 | 0.06 | 2.27 (2.02 - 2.55) | <0.001 |
|  | Mastiff Like | 789 (6.5) | 19339 (12.2) | -0.03 | 0.058 | 0.97 (0.87 - 1.09) | 0.601 |
|  | Scent Hound | 178 (1.5) | 3889 (2.5) | 0.134 | 0.089 | 1.14 (0.96 - 1.36) | 0.132 |
|  | Sight Hound | 112 (0.9) | 2456 (1.6) | 0.144 | 0.11 | 1.16 (0.93 - 1.43) | 0.188 |
|  | Small Terriers | 1967 (16.2) | 17092 (10.8) | 1.047 | 0.047 | 2.85 (2.6 - 3.12) | <0.001 |
|  | Spaniel | 1464 (12) | 18705 (11.8) | 0.641 | 0.048 | 1.90 (1.73 - 2.09) | <0.001 |
|  | Toy | 1132 (9.3) | 11150 (7.1) | 0.848 | 0.052 | 2.34 (2.11 - 2.58) | <0.001 |
|  | Unclassified | 1462 (12) | 21774 (13.8) | 0.549 | 0.048 | 1.73 (1.58 - 1.9) | <0.001 |
|  | Working Dog | 358 (2.9) | 6124 (3.9) | 0.334 | 0.07 | 1.40 (1.22 – 1.60) | <0.001 |
| IMD | 1 (most deprived) | 1093(10) | 9095(6) |  |  | 1.00 | - |
|  | 2 | 956(9) | 9798(7) | -0.253 | 0.048 | 0.78 (0.71 - 0.85) | <0.001 |
|  | 3 | 1043(9) | 10604(7) | -0.214 | 0.048 | 0.81 (0.74 - 0.89) | <0.001 |
|  | 4 | 1186(11) | 13362(9) | -0.363 | 0.047 | 0.7 (0.63 - 0.76) | <0.001 |
|  | 5 | 1225(11) | 14862(10) | -0.407 | 0.047 | 0.67 (0.61 - 0.73) | <0.001 |
|  | 6 | 1282(12) | 16596(12) | -0.48 | 0.047 | 0.62 (0.56 - 0.68) | <0.001 |
|  | 7 | 1216(11) | 17447(12) | -0.55 | 0.048 | 0.58 (0.53 - 0.63) | <0.001 |
|  | 8 | 1168(11) | 16755(12) | -0.541 | 0.048 | 0.58 (0.53 - 0.64) | <0.001 |
|  | 9 | 978(9) | 16616(12) | -0.68 | 0.05 | 0.51 (0.46 - 0.56) | <0.001 |
|  | 10 (least deprived) | 833(8) | 16697(12) | -0.847 | 0.053 | 0.43 (0.39 - 0.48) | <0.001 |
| **Continuous Variables** | | | | | | | |
| Age |  |  |  | 0.115 | 0.022 | 1.12 (1.08 - 1.17) | <0.001 |
|  |  |  |  | 0.058 | 0.015 | 1.06 (1.03 - 1.09) | <0.001 |
|  |  |  |  | -0.073 | 0.011 | 0.93 (0.91 - 0.95) | <0.001 |

# Supplementary Table 4 – Risk of veterinary-recorded flea infestation in cats (N=22,276) for England Only with IMD . ^a^ Standard Error, ^b^ Odds Ratio and ^c^ 95% Confidence Interval

| **Variable** | **Level** | **Case (%)** | **Controls (%)** | **beta** | **SE^a^** | **OR^B^ (95% CI^C^)** | **P** |
| --- | --- | --- | --- | --- | --- | --- | --- |
|  | (Intercept) |  |  | -3.843 | 0.113 | - | - |
| **Categorical Variables** | | | | | | | |
| Sex + Neuter | Male Entire | 3264 (26.8) | 31029 (19.7) | - | - | 1.00 | - |
|  | Male Neutered | 3122 (25.7) | 49660 (31.4) | -0.476 | 0.029 | 0.62 (0.59 - 0.66) | <0.001 |
|  | Female Entire | 2875 (23.6) | 25965 (16.4) | -0.045 | 0.034 | 0.96 (0.9 - 1.02) | 0.181 |
|  | Female Neutered | 2906 (23.9) | 51249 (32.5) | -0.493 | 0.029 | 0.61 (0.58 - 0.65) | <0.001 |
| Months | January | 726 (6) | 13927 (8.8) | - | - | 1.00 | - |
|  | February | 650 (5.3) | 13353 (8.5) | -0.148 | 0.042 | 0.86 (0.8 - 0.94) | <0.001 |
|  | March | 453 (3.7) | 13125 (8.3) | -0.266 | 0.044 | 0.77 (0.7 - 0.84) | <0.001 |
|  | April | 418 (3.4) | 11122 (7) | -0.347 | 0.047 | 0.71 (0.65 - 0.78) | <0.001 |
|  | May | 439 (3.6) | 11631 (7.4) | -0.442 | 0.047 | 0.64 (0.59 - 0.71) | <0.001 |
|  | June | 656 (5.4) | 12764 (8.1) | -0.261 | 0.044 | 0.77 (0.71 - 0.84) | <0.001 |
|  | July | 1406 (11.6) | 14963 (9.5) | 0.085 | 0.039 | 1.09 (1.01 - 1.18) | 0.028 |
|  | August | 1698 (14) | 13970 (8.8) | 0.211 | 0.038 | 1.24 (1.15 - 1.33) | <0.001 |
|  | September | 1846 (15.2) | 13638 (8.6) | 0.317 | 0.038 | 1.37 (1.28 - 1.48) | <0.001 |
|  | October | 1708 (14) | 14145 (9) | 0.287 | 0.037 | 1.33 (1.24 - 1.43) | <0.001 |
|  | November | 1288 (10.6) | 13061 (8.3) | 0.255 | 0.038 | 1.29 (1.2 - 1.39) | <0.001 |
|  | December | 879 (7.2) | 12204 (7.7) | 0.051 | 0.041 | 1.05 (0.97 - 1.14) | 0.207 |
| Rural/Urban | Rural | 3515 (28.9) | 51184 (32.4) | - | - | 1.00 | - |
|  | Urban | 8653 (71.1) | 106719 (67.6) | 0.133 | 0.024 | 1.14 (1.09 - 1.2) | <0.001 |
| NUTS1 | **UKC** (North East) | 704 (5.8) | 14337 (9.1) |  |  | 1.00 | - |
|  | UKD (North West) | 1328 (10.9) | 16167 (10.2) | 0.433 | 0.141 | 1.05 (0.97 - 1.14) | 0.207 |
|  | UKE (Yorks & Humber) | 1157 (9.5) | 17843 (11.3) | 0.261 | 0.146 | 1.54 (1.17 - 2.03) | 0.002 |
|  | UKF (East Midlands) | 637 (5.2) | 9797 (6.2) | 0.292 | 0.148 | 1.3 (0.98 - 1.73) | 0.073 |
|  | UKG (West Midlands) | 699 (5.7) | 11937 (7.6) | 0.264 | 0.151 | 1.34 (1 - 1.79) | 0.048 |
|  | UKH (East of England) | 1691 (13.9) | 19295 (12.2) | 0.356 | 0.144 | 1.3 (0.97 - 1.75) | 0.08 |
|  | UKI (Greater London) | 110 (0.9) | 1708 (1.1) | 0.536 | 0.173 | 1.43 (1.08 - 1.89) | 0.013 |
|  | UKJ (South East) | 2763 (22.7) | 32446 (20.5) | 0.539 | 0.131 | 1.71 (1.22 - 2.4) | 0.002 |
|  | UKK (South West) | 1891 (15.5) | 18307 (11.6) | 0.693 | 0.141 | 1.71 (1.32 - 2.22) | <0.001 |
| Breed | West Europe | 122 (1) | 2041 (1.3) | - | - | 1.00 | - |
|  | Crossbreed | 3322 (27.3) | 33811 (21.4) | 0.332 | 0.107 | 0.56 (0.48 - 0.66) | <0.001 |
|  | Mediterranean | 619 (5.1) | 6823 (4.3) | 0.876 | 0.044 | 1.18 (1.09 - 1.27) | 0.002 |
|  | Unclassified | 642 (5.3) | 14699 (9.3) | 0.819 | 0.06 | 0.82 (0.33 - 2.08) | <0.001 |
|  | Asian | 789 (6.5) | 19339 (12.2) | -0.03 | 0.058 | 0.75 (0.65 - 0.85) | <0.001 |
| IMD | 1 (most deprived) | 1610(8) | 3235(5) |  |  | 1.00 | - |
|  | 2 | 1833(9) | 3866(6) | -0.084 | 0.043 | 0.92 (0.84 - 1) | 0.053 |
|  | 3 | 1874(9) | 4408(7) | -0.187 | 0.043 | 0.83 (0.76 - 0.9) | <0.001 |
|  | 4 | 2243(11) | 5583(9) | -0.239 | 0.042 | 0.79 (0.73 - 0.86) | <0.001 |
|  | 5 | 2138(10) | 6329(10) | -0.369 | 0.042 | 0.69 (0.64 - 0.75) | <0.001 |
|  | 6 | 2135(10) | 7099(12) | -0.495 | 0.042 | 0.61 (0.56 - 0.66) | <0.001 |
|  | 7 | 2364(11) | 7192(12) | -0.38 | 0.042 | 0.68 (0.63 - 0.74) | <0.001 |
|  | 8 | 2143(10) | 7330(12) | -0.505 | 0.042 | 0.60 (0.56 - 0.66) | <0.001 |
|  | 9 | 2106(10) | 7528(12) | -0.532 | 0.042 | 0.59 (0.54 - 0.64) | <0.001 |
|  | 10 (least deprived) | 2215(11) | 8725(14) | -0.621 | 0.043 | 0.54 (0.49 - 0.58) | <0.001 |
| **Continuous Variables** | | | | | | | |
| Age |  |  |  | 0.115 | 0.022 | 1.12 (1.08 - 1.17) | <0.001 |
|  |  |  |  | 0.058 | 0.015 | 1.06 (1.03 - 1.09) | <0.001 |
|  |  |  |  | -0.073 | 0.011 | 0.93 (0.91 - 0.95) | <0.001 |
